# Identification of pararosaniline as a modifier of RNA splicing in *Caenorhabditis elegans*

**DOI:** 10.1093/g3journal/jkad241

**Published:** 2023-10-19

**Authors:** Dylan Huynh, Cheng-Wei Wu

**Affiliations:** Department of Veterinary Biomedical Sciences, Western College of Veterinary Medicine, University of Saskatchewan, Saskatoon, SK S7N 5B4, Canada; Department of Veterinary Biomedical Sciences, Western College of Veterinary Medicine, University of Saskatchewan, Saskatoon, SK S7N 5B4, Canada; Toxicology Centre, University of Saskatchewan, Saskatoon, SK S7N 5B3, Canada; Department of Biochemistry, Microbiology and Immunology, College of Medicine, University of Saskatchewan, Saskatoon, SK S7N 5E5, Canada

**Keywords:** toxcast, RNA splicing, *C. elegans*, pararosaniline, aging, stress

## Abstract

Posttranscriptional splicing of premessenger RNA (mRNA) is an evolutionarily conserved eukaryotic process for producing mature mRNA that is translated into proteins. Accurate splicing is necessary for normal growth and development, and aberrant splicing is increasingly evident in various human pathologies. To study environmental factors that influence RNA splicing, we employed a fluorescent *Caenorhabditis elegans* in vivo splicing reporter as a biomarker for splicing fidelity to screen against the US EPA ToxCast chemical library. We identified pararosaniline hydrochloride as a strong modifier of RNA splicing. Through gene expression analysis, we found that pararosaniline activates the oxidative stress response and alters the expression of key RNA splicing regulator genes. Physiological assays show that pararosaniline is deleterious to *C. elegans* development, reproduction, and aging. Through a targeted RNAi screen, we found that inhibiting protein translation can reverse pararosaniline's effect on the splicing reporter and provide significant protection against long-term pararosaniline toxicity. Together, this study reveals a new chemical modifier of RNA splicing and describes translation inhibition as a genetic mechanism to provide resistance.

## Introduction

Premessenger RNA (mRNA) splicing is a highly regulated and essential posttranscriptional process that splices out noncoding introns to produce a mature mRNA destined for protein translation (Nilsen 2003). Accurate splicing of RNA molecules is required to maintain cellular homeostasis, and alternative splicing of transcripts contributes to proteome diversity that is critical in coordinating organismal growth and development (Nilsen and Graveley 2010). Conversely, emerging evidence suggests that defects in RNA splicing metabolism are associated with a growing list of human diseases including neurodegeneration and cancer (Montes et al. 2019). The decline in RNA splicing fidelity has also been shown as a factor in aging, likely contributing to the progressive onset of genomic instability and loss in protein homeostasis (Bhadra et al. 2020). To date, factors that contribute to defects in RNA splicing metabolism remain poorly understood.

**Fig. 1.**
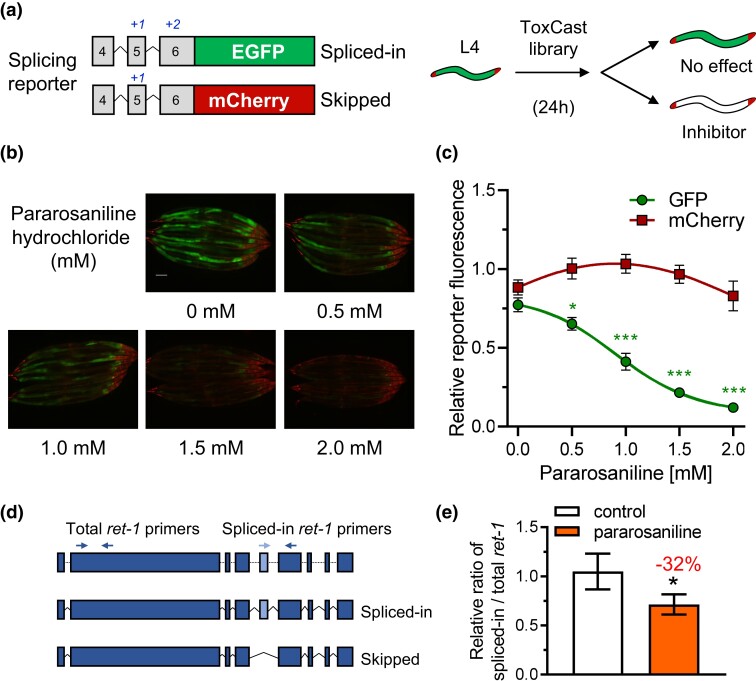
Identification of pararosaniline as a modifier of RNA splicing. a) Schematic of the in vivo RNA splicing reporter and the chemical screen workflow. b) Representative fluorescent images and c) relative GFP and mCherry fluorescence of the splicing reporter worm strain exposed to 0–2 mM of pararosaniline on solid media. The scale bar is 100 µm. The data point in c) indicates mean ± SEM of *n* = 9 groups of 8 worms/group. d) qPCR primer design and e) relative expression level of total and spliced-in *ret-1* endogenous transcript in N2 worms under control or exposure to 1 mM of pararosaniline on solid media. The bar graph indicates mean ± SD of *n* = 4 groups of ∼300–500 worms/group per condition. *P < 0.05, **P < 0.01, and ***P < 0.001 as determined by 1-way ANOVA in c) and student's *t*-test in e).

Recently, we identified in the *Caenorhabditis elegans* model that exposure to the heavy metal cadmium can alter transcriptome-wide RNA splicing, suggesting aberrant exposure to environmental contaminants as a deleterious modifier of RNA splicing metabolism (Wu et al. 2019). However, most chemicals in our environment are untested and uncharacterized, which can pose a risk to human and animal health in the context of exposure to chemicals with limited or unknown toxicological properties. Here, we employed a transgenic *C. elegans* strain expressing a fluorescent alternative splicing reporter that serves as a biomarker for RNA splicing fidelity. In brief, the in vivo splicing reporter is constructed with a pair of *ret-1* minigenes driven by the ubiquitous *eft-3* promoter and fused to either a GFP or mCherry reporter (Kuroyanagi et al. 2013). When exon 5 of the *ret-1* minigene is spliced-in, the GFP reporter is activated; conversely, when exon 5 is skipped, the GFP signal is lost and the mCherry reporter is activated (Fig. 1a). Previous studies using this splicing reporter have shown that the GFP reporter becomes inactivated when splicing fidelity is altered via aging or cadmium exposure (Heintz et al. 2017; Chomyshen et al. 2022). Using this splicing reporter, we screened against the complete US EPA ToxCast library composed of 4,665 chemicals to identify compounds that can alter the GFP signal of the splicing reporter, as a means to identify chemicals with potential toxicity to modify splicing fidelity.

In this study, we identified a common industrial dye reagent pararosaniline as a modifier of RNA splicing metabolism that has broad toxicity against *C. elegans* developmental, reproductive, and aging physiology. Through gene expression analysis, we found that exposure to pararosaniline activates the oxidative stress response and alters the expression of select genes that function in RNA metabolism. Using a targeted RNAi screen, we found that inhibiting protein translation can suppress pararosaniline's effect on RNA splicing and protect against long-term toxicity. Overall, our results reveal a new environmental compound that alters RNA splicing in *C. elegans* and identified translation inhibition as a genetic modifier of this response.

## Materials and methods

### *C. elegans* culture

This study used the following strains: N2 Bristol wild-type, MWU85*lin-15 (n765) ybIs2167* [*eft-3::RET-1E4E5(+1)E6-GGS6-mCherry eft-3::RET-1E4E5(+1)E6-(+2)GGS6-EGFP lin-15 (+) pRG5271Neo*] *X; col-121(nx3)*, WBM535*wbmEx237* [*pCH11 (eft-3::ret-1E4E5(+1)E6-(+2)-GGS6-mCherry) + pCH12 (eft-3::ret-1E4E5(+1)E6-GGS6-EGFP)*], MWU222*cwwEx52* [*snr-2p::SNR-2; rol-6(su1006)*]; *ybIs2167*. The MWU222 SNR-2 overexpression strain was created by microinjection of a 1465 bp of PCR amplified DNA fragment that contained the genomic sequence of *snr-2* flanked by its native 5′ promoter and 3′ downstream elements. All strains were cultured on nematode growth medium (NGM) agar plates using standard conditions (Brenner 1974).

### Chemical screen for RNA splicing modulators

The MWU85 strain was age synchronized with hypochloride treatment and grown to 1-day-old adults on NA22-seeded NGM plates. Approximately 50–75 adult worms were transferred to each well of a 96-well microplate and incubated with a chemical from the ToxCast EPA compound library at a final concentration of 200 µM in 1% DMSO for 24 h. Select compounds were tested at 50–300 μM due to concentration differences of the chemical stock ranging from 5 to 30 mM. After 24 h, the wells were screened manually using an Olympus SZX16 microscope for a reduction in GFP fluorescence. Chemicals identified in the screening were then rescreened over a range of chemical doses on solid media for confirmation.

### *C. elegans* microscopic imaging

Worms were imaged either by mounting on a glass microscope slide with a Zeiss Axioskop 50 microscope fitted with a QImaging Retiga R3 camera or in a microplate using an Agilent Cytation-5 Cell Imaging Multi-Mode Reader. In the microscope slide method, worms were anesthetized with 1% sodium azide on a 2% agarose pad followed by fluorescent imaging. In the Cytation-5 method, worms were incubated in M9 buffer and anesthetized with 5 mM of levamisole.

### *C. elegans* physiological assays

To assess the chemical effects on the development of *C. elegans*, the N2 strain was cultured, age synchronized, and transferred at the L1 larval stage to *E. coli-*seeded NGM plates containing 0, 0.5, 1, 1.5, and 2 mM of pararosaniline hydrochloride. A 0.8% DMSO only was used in the 0 mM control representing the DMSO concentration in the 2 mM exposure, the highest concentration tested. The plates were incubated for 48 h at 20°C, and the worms were imaged directly on their plates with an Olympus SZX16 microscope fitted with a QImaging Retiga R3 camera. The *C. elegans* body length was quantified using ImageJ to assess development. Three independent development assays were performed with a total of *n* = 100 worms per condition.

To assess the chemical effects on *C. elegans* reproduction, the N2 strain was age synchronized and grown to the L4 young adult larval stage on *E. coli-*seeded NGM plates. To initiate the assay and chemical exposure, a single worm was transferred to an *E. coli-*seeded NGM plate containing 0 (0.8% DMSO), 0.5, 1, 1.5, and 2 mM of pararosaniline hydrochloride. The number of offspring including eggs laid and hatched progenies was recorded daily followed by moving the same worm to a new plate with identical chemical concentration. This process was repeated until reproduction had ceased. The total number of offspring and eggs were tallied over the reproductive period to obtain the total brood size. Three independent reproduction assays were performed with a total of 27–39 worms scored for 0–1.5 mM of pararosaniline. For the 2 mM pararosaniline, only a total of 7 worms were scored due to the high mortality rate observed at this concentration during the reproduction window.

To assess the chemical effects on *C. elegans* aging, the N2 strain was age synchronized and grown to the L4 young adult larval stage on NGM plates seeded with empty vector (EV) RNAi (L4440) bacteria containing 1 mM isopropyl β-D-thiogalactopyranoside (IPTG) and 0.05 mg/mL carbenicillin. To initiate lifespan assay in the presence of chemicals, approximately 40 worms were transferred to NGM plates seeded with EV bacteria containing 0 (0.8% DMSO), 0.5, 1, 1.5, and 2 mM of pararosaniline hydrochloride. The plates were incubated at 20°C, and the worms were transferred to identical new plates daily until reproduction had ceased. This was done to prevent the mixing of the offspring with the parental worms which are indistinguishable after adulthood is reached. The deaths were counted daily until no worms were remaining on the plate. Worms were considered dead if they did not respond to a gentle tap with a flame sterilized platinum pick. Worms were censored if they exhibited any vulval or intestinal defects (protrusions) or if they died from drying out on the sidewall of the plate. Three independent lifespan assays were performed with a total of *n* = 86–146 worms scored per condition. For lifespan involving RNAi knockdown, RNAi was initiated at day 1 of adulthood for 48 h on control plates followed by transfer to NGM plates seeded with corresponding RNAi containing 0, 0.5, 1, 1.5, and 2 mM of pararosaniline hydrochloride. Three independent RNAi lifespan assays were performed with a total of *n* = 70–152 worms per condition.

### RNA extraction and qPCR

Methods used for gene expression analysis were followed as previously described in detail (Tabarraei et al. 2023). Briefly, age synchronized 1 day of worms were incubated on NGM agar plates with 1 mM of pararosaniline or 0.4% DMSO only control for 24 h. RNA was extracted from *n* = 4 biological replicates each containing ∼300–500 worms per condition using the Purelink RNA mini kit (12183020; ThermoFisher). RNA was then treated with DNaseI (EN0521; ThermoFisher) followed by cDNA synthesis using the Invitrogen Multiscribe reverse transcriptase (4311235; ThermoFisher) with the Applied Biosystems ProFlex Thermocycler. Real-time PCR (qPCR) was performed using the Quanstudio3 system with the PowerUp SYBR Green Master Mix (#A25741). Primers used in this study are listed in Supplementary Table 1.

### RNAi screen

A targeted RNAi knockdown screen was conducted to identify the genes that when knocked down, protect against RNA splicing errors after chemical exposure. The screen included selectively knocking down 74 different genes that were previously found in a previous study to protect against RNA splicing errors induced by cadmium exposure, as evident by the retention of the in vivo GFP fluorescence of the splicing reporter following cadmium exposure (Chomyshen et al. 2022). Age-synchronized MWU85 worms at the L1 larval stage were incubated with concentrated RNAi bacteria in NGM buffer containing 1 mM IPTG and 0.05 mg/mL carbenicillin in a 96-well microplate. After 48 h of incubation at 20°C, pararosaniline hydrochloride was added at a final concentration of 200 μM in liquid media, and the microplate was incubated for an additional 24 h followed by screening for changes in GFP fluorescence of the in vivo splicing reporter. A 1% DMSO only was used as a negative control. The top 10 genes with high levels of GFP fluorescence following chemical exposure identified qualitatively were selected for a secondary screen with the Cytation-5 system.

### Statistical analyses

GraphPad Prism 8.2.1 was used to create graphs and perform statistical analyses. The mean values are shown with error bars indicating the SD. A 1-way ANOVA was used to compare 3 or more groups corrected for multiple comparisons using the Dunnett method to determine statistical significance for the dose-response exposures as well as the development and reproduction assays. OASIS 2 was used to analyze the lifespan assays including the mean lifespan and statistical significance using a log-rank test (Han et al. 2016). A student's *t*-test was used to compare 2 groups corrected for multiple comparisons using the Holm–Sidak method to determine the statistical significance of the qPCR results. Statistical significance was indicated with the following: *P ≤ 0.05, **P ≤ 0.01, ***P ≤ 0.001, ****P ≤ 0.0001, and ns = not significant.

## Results

### Pararosaniline exposure alters RNA splicing

We previously identified cadmium as a potential environmental contaminant that alters RNA splicing metabolism in *C. elegans* (Wu et al. 2019; Chomyshen et al. 2022). To further our understanding of other environmental chemicals that may influence RNA splicing, we performed a large-scale chemical screen using the ToxCast compound library with the *C. elegans* in vivo RNA splicing reporter (Fig. 1a) (Richard et al. 2016). To reduce the chemical concentration used for the screen, we first introduced the *col-121(nx3)* allele into the splicing reporter which carries a collagen gene mutation that increases cuticle permeability to facilitate peripheral chemical uptake (Shin et al. 2019). From the primary chemical screen, we identified 15 chemicals that caused a reduction of the GFP fluorescence of the RNA splicing reporter (Supplementary Table 2). We found that exposure to 200 µM of pararosaniline, also known as Basic Red 9, led to the strongest reduction of the GFP signal of the splicing reporter, which indicated a decrease in the abundance of the fully spliced-in *ret-1* minigene. We decided to focus on characterizing pararosaniline for the remainder of the study given it led to the strongest phenotype. We next performed a dose range test using solid media and found that a higher concentration of pararosaniline was required to reduce the GFP signal, likely due to a variance between liquid and solid media for chemical uptake. A significant decrease in GFP signal could be observed starting at 0.5 mM of pararosaniline; interestingly, the mCherry fluorescence did not significantly alter across the range of exposure (Fig. 1, b and c). The lack of changes to mCherry fluorescence may be due to the preferential expression of this reporter in the muscle and nervous system where there could be a difference in chemical uptake and bioavailability compared with the intestine where the GFP reporter is primarily expressed. To determine if the reduction of GFP signals caused by pararosaniline could be linked to a possible variability in fluorophore stability between GFP and mCherry, we utilized an inverted splicing reporter where mCherry is fused to the spliced-in variant and GFP is fused to the skipped variant (Supplementary Fig. 1a; Heintz et al. 2017). Using the inverted reporter, we found that exposure to pararosaniline decreased mCherry fluorescence without affecting the GFP signal, which is consistent with our initial observation that pararosaniline induces a decrease in the spliced-in variant of the *ret-1* transcript (Supplementary Fig. 1, b and c).

To determine that pararosaniline affected endogenous *ret-1* splicing, we measured the relative abundance of total and spliced-in *ret-1* transcript abundance via qPCR in N2 worms exposed to 1 mM of pararosaniline on solid media. We chose a concentration of 1 mM since it led to a significant loss of GFP fluorescence with no mortality observed after 24 h of exposure. The total *ret-1* transcript was measured using primer pairs that bind to exon 2 of the gene which is unaffected by alternative splicing; meanwhile, the spliced-in *ret-1* transcript was measured using primers that bind to exon 5 and 6 of the transcript, with exon 5 not detected in the skipped variant of *ret-1* (Fig. 1d). We found that exposure to pararosaniline decreases the relative ratio of spliced-in/total *ret-1* transcript by 32% (Fig. 1e).

To consider the possibility that pararosaniline may act as a transcriptional inhibitor of gene expression in the intestine, we tested the effects of pararosaniline exposure on the expression of several intestine-specific genes (Mcghee et al. 2007). Of the 11 intestine-specific genes tested, pararosaniline only led to a slight decrease in the expression of *vit-6*, suggesting that this chemical does not broadly inhibit transcription of intestinal gene expression (Supplementary Fig. 2). Together, our results here identified pararosaniline as a chemical modifier of RNA splicing in *C. elegans*.

### Exposure to pararosaniline is deleterious to *C. elegans* physiology

**Fig. 2.**
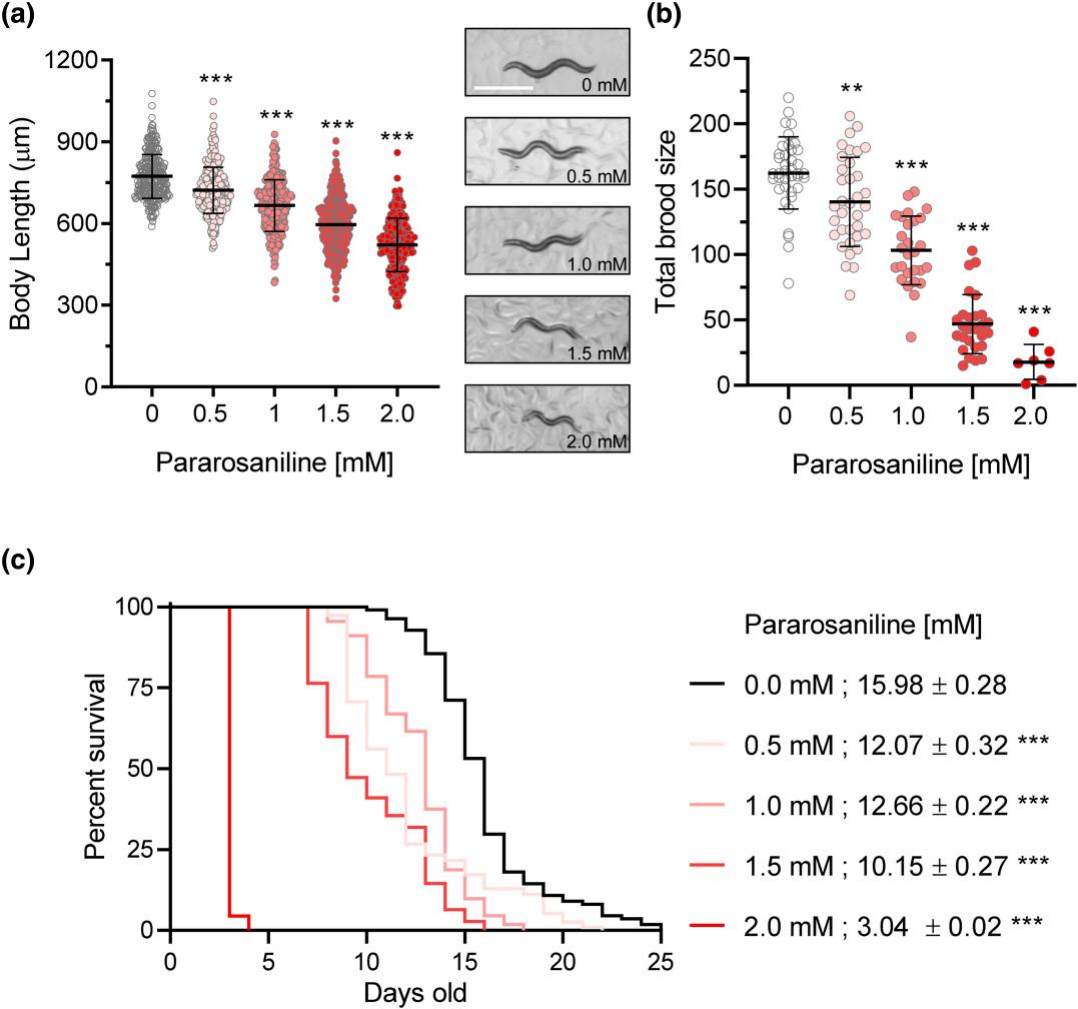
Pararosaniline is deleterious to *C. elegans* physiology. Effects of 0–2 mM pararosaniline exposure in solid media on *C. elegans* a) development, b) brood size, and c) lifespan. Mean lifespan ± SE is presented. The scale bar in a) is 1000 µm. For the development assay, combined data from 3 total trials with 101 worms measured per trial at each concentration are shown. For brood size assay, 3 independent reproduction assays were performed with a total of 27–39 worms scored for 0–1.5 mM of pararosaniline. For the 2 mM pararosaniline, a total of 7 worms were scored due to the high lethality at this concentration. For lifespan assay, 3 trials were performed with at least 90 animals scored per condition in each trial. Trial 1 is shown, and detailed statistic of the lifespan trial is presented in Supplementary Table 3. *P < 0.05, **P < 0.01, and ***P < 0.001 as determined by 1-way ANOVA in a) and b) and the long-rank test in c). Error bars indicate ± SD.

To characterize the effects of pararosaniline on *C. elegans* physiology, we measured how exposure to increasing concentrations of pararosaniline affects *C. elegans* development, reproduction, and lifespan. We performed these assays on solid media given that this is the most common methodology for these assays; as such, we used pararosaniline concentrations ranging from 0 (control) to 2 mM which was shown to affect the splicing reporter in a dose response manner (Fig. 1, b and c). At all concentrations tested, we found that exposing worms starting at the L1 stage caused a significant decrease in body length when assessed after 48 h (Fig. 2a). Similarly, exposure to pararosaniline starting at the L4 stage significantly reduced *C. elegans* brood size across all concentrations tested (Fig. 2b). It should be noted that only 7 worms across 3 trials were scored at the highest concentration of 2.0 mM due to the high incidence of mortality observed. We then tested the effects of growing worms continuously on pararosaniline starting at the L4 stage to determine its impact on aging. Consistently, we saw a significant decrease in worm lifespan across all concentrations tested (Fig. 2c). Together, these results show that exposure to pararosaniline has broad deleterious effects on *C. elegans* physiology in a dose-dependent manner.

### Gene expression alteration in response to pararosaniline

**Fig. 3.**
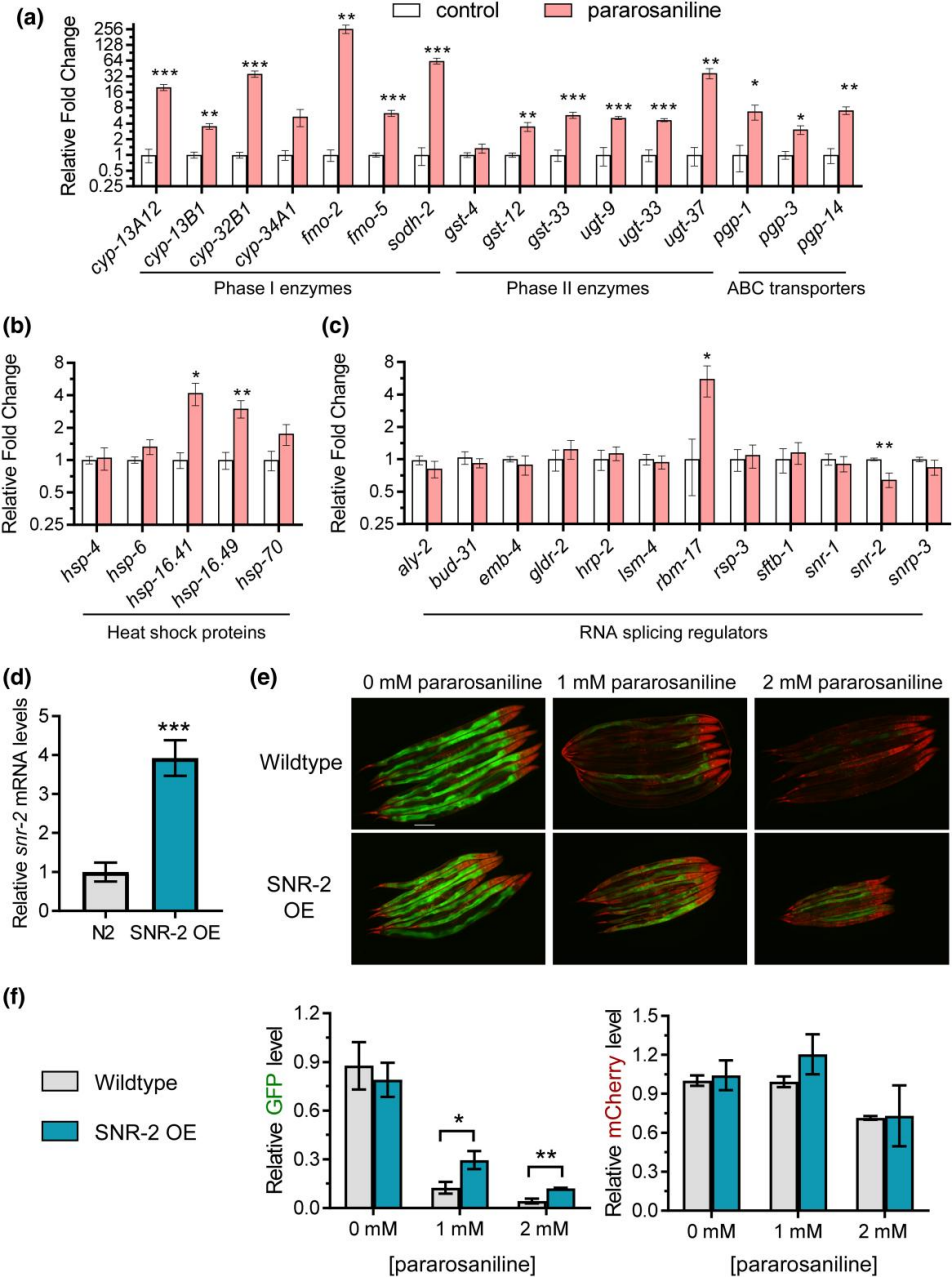
Transcriptional response to pararosaniline and effects of *snr-2* overexpression. Relative expression of a) xenobiotic detoxification genes, b) heat shock protein genes, and c) RNA splicing regulator genes after 24 h exposure to 1 mM of pararosaniline on solid media. d) Relative expression levels of *snr-2* in an SNR-2 overexpression strain. e) Representative fluorescent micrograph and f) quantification of the splicing reporter in wild-type and SNR-2 overexpression worms treated with 0–2 mM of pararosaniline. The data point in f) indicates mean ± SD of *n* = 3 groups of 8 worms/group. *P < 0.05, **P < 0.01, and ***P < 0.001 as determined by the student's *t*-test corrected for multiple comparisons using the Holm–Sidak method.

As exposure to pararosaniline was broadly deleterious to *C. elegans* physiology, we next investigated the effect of this chemical on the expression of stress responsive genes involved in xenobiotic detoxification and proteome stability. Worms were treated with 1 mM of pararosaniline for 24 h on solid media, the same regimen that altered *ret-1* splicing (Fig. 1e) and impaired *C. elegans* physiology (Fig. 2). We observed that 14 of 16 genes that function within the 3 phases of xenobiotic detoxification pathways were significantly upregulated (Fig. 3a). This suggested that pararosaniline exposure likely induced oxidative stress given the strong activation of xenobiotic detoxification genes. Of the 5 heat shock protein genes measured, *hsp-16.41* and *hsp-16.49* were slightly elevated, suggesting that pararosaniline exposure may also influence proteome stability (Fig. 3b).

Given that pararosaniline altered the RNA splicing reporter, we next sought to determine whether this chemical influenced the expression of select genes involved in RNA splicing metabolism. We observed that pararosaniline exposure caused the upregulation of the *rbm-17* gene which is predicted to function as an RNA binding protein within the spliceosomal complex (Fig. 3c). Interestingly, we also observed a significant downregulation of the *snr-2* gene, which encodes a small nuclear ribonucleoprotein that functions as a core spliceosomal factor (Fig. 3c). In correlation, a previous study has shown that the knockdown of *snr* encoding genes led to a loss of the GFP signal of the splicing reporter, suggesting that reduced expression of *snr-2* could lead to changes in RNA splicing dynamic (Heintz et al. 2017). To explore the hypothesis that pararosaniline alters the splicing reporter by decreasing the expression of *snr-2*, we overexpressed *snr-2* in the splicing reporter to test for its potential to provide resistance against pararosaniline (Fig. 3d). We found that pararosaniline exposure still resulted in a decrease in the GFP signal of the splicing reporter in worms overexpressing *snr-2* relative to the 0 mM control; however, there was significant retention of GFP signal in the *snr-2* overexpression strain compared with the wildtype after exposure to 1 and 2 mM of paraosaniline (Fig. 3, e and f). This suggested that overexpression of *snr-2* can provide partial resistance against pararosaniline-induced changes to the splicing reporter. Together, these results show that pararosaniline exposure activates the oxidative stress response as well as alters the expression of RNA splicing regulator genes and that the decrease in *snr-2* expression may serve as a partial mechanism through which pararosaniline affects RNA splicing.

### Translation inhibition protects against pararosaniline toxicity

**Fig. 4.**
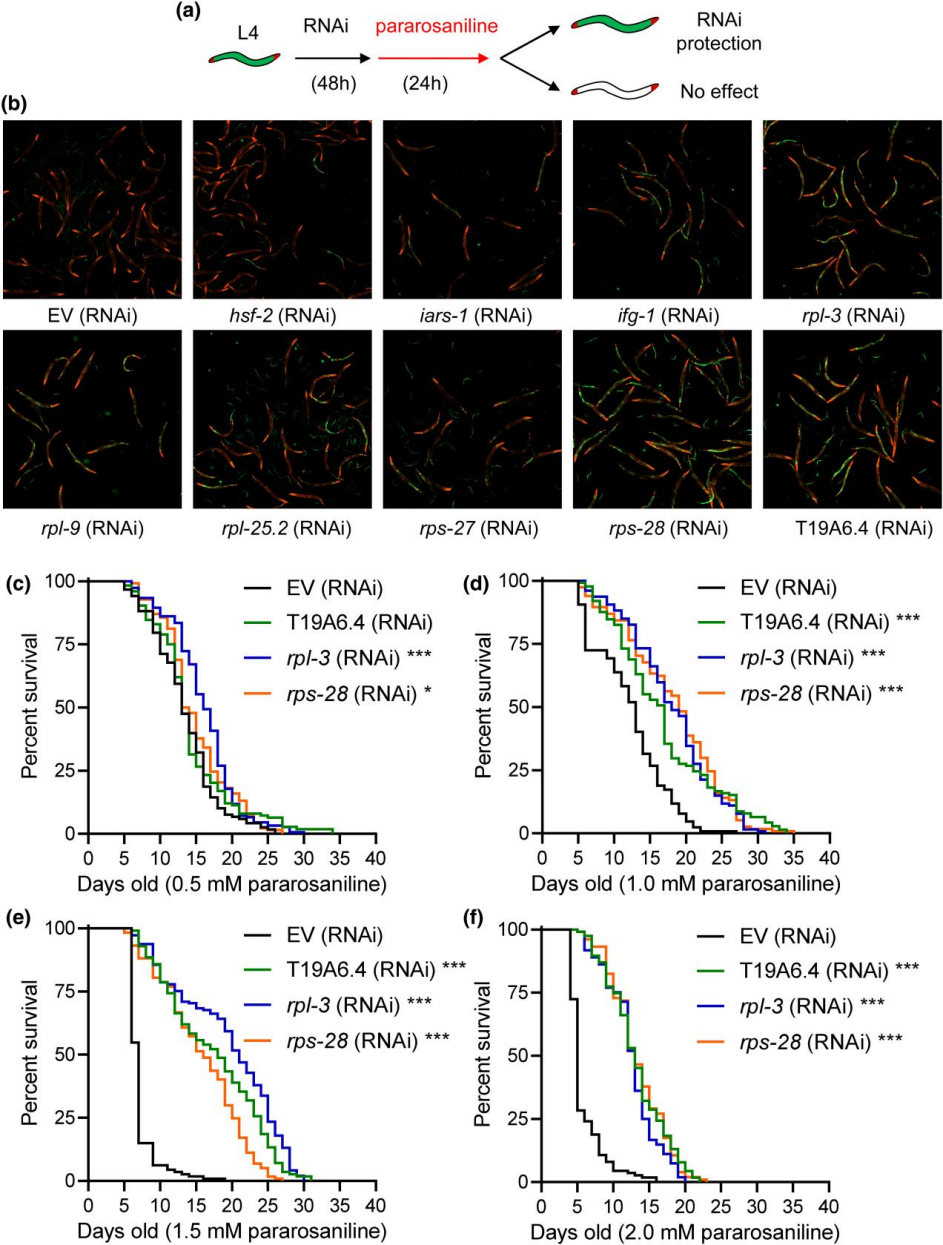
Translation inhibition suppresses pararosaniline toxicity. a) Schematic of targeted RNAi library screen for suppressor of pararosaniline's effect on the in vivo splicing reporter. b) Representative fluorescent images showing effects of candidate gene knockdowns on the splicing reporter after exposure to 200 µM of pararosaniline in liquid media for 24 h. c) Effects of T19A6.4, *rpl-3*, and *rps-28* gene knockdown on *C. elegans* survival to 0.5–2 mM of pararosaniline on solid media. *P < 0.05, **P < 0.01, and ***P < 0.001 as determined by the log-rank test. Three independent trials were performed with trial 1 shown. Detailed statistics are shown in Supplementary Table 3.

We previously identified from a genome-wide RNAi screen that the knockdown of genes primarily functioning in protein translation inhibited cadmium-induced splicing alterations (Chomyshen et al. 2022). To determine if gene knockdowns that inhibited cadmium-induced alternative splicing could exert similar effects on pararosaniline toxicity, we rescreened the 74 candidate genes previously identified to test their effects of pararosaniline-induced splicing (Fig. 4a). Consistently, we found that RNAi knockdown of translation related genes including *ifg-1*, *rpl-3*, *rpl-9*, *rpl-25.2*, *rps-27*, and *rps-28* preserved the GFP fluorescence of the splicing reporter after pararosaniline exposure (Fig. 4b). Knockdown of T19A6.4, which encodes a homolog of the human transmembrane protein 144, also led to strong retention of GFP signal post pararosaniline exposure (Fig. 4b). We next chose the top 3 genes that when knocked down led to the strongest GFP retention of the splicing reporter, T19A6.4, *rpl-3*, *rps-28*, and tested for their effects on pararosaniline survival. At the lowest dose of 0.5 mM, knockdown of *rpl-3* and *rps-28* mildly extended *C. elegans* survival (Fig. 4c). From exposure doses of 1–2 mM, knockdown of all 3 candidate genes strongly extended *C. elegans* survival compared with the EV control (Fig. 4d–f). Together, these results show that the knockdown of genes functioning in protein translation inhibits pararosaniline-induced splicing alternations and protects against chronic toxicity.

## Discussion

RNA splicing is a critical posttranscriptional process that contributes to transcriptome homeostasis and proteome diversity (Will and Lührmann 2011). In recent years, aberrant modifications to RNA splicing fidelity have emerged as a factor in accelerated aging and other pathological states including cancer and neurodegeneration (Scotti and Swanson 2015; Bhadra et al. 2020). In this study, we identified pararosaniline as a modifier of RNA splicing in *C. elegans*. Pararosaniline is a triarylmethane dye that is commonly used for microbial staining and as an industrial agent in a wide variety of commercial products including paper, textiles, cosmetics, and paint (Shetty et al. 2023). Pararosaniline hydrochloride was tested for carcinogenicity in mice and rats by oral administration where it induced hepatocellular carcinomas and liver tumors in mice and significantly increased the incidences of benign and malignant tumors in rats (Huff et al. 1989). While environmental levels of pararosaniline are not regularly investigated, its recent emergence as a pharmacologically active chemical has led to the development of analytical techniques for detection in water and air samples (Kowalska and Jeżewska 2019; Tkaczyk-Wlizło et al. 2022). Our study finds that exposure to pararosaniline alters RNA splicing as determined by using an in vivo splicing reporter (Fig. 1; Supplementary Fig. 1) and that this chemical is deleterious to multiple facets of *C. elegans* physiology (Fig. 2) potential via inducing oxidative stress and altered expression of splicing regulators (Fig. 3). Through a targeted genetic screen, we show that suppression of protein translation can resist pararosaniline's effect on the RNA splicing reporter and provide protection against chronic toxicity to this chemical (Fig. 4).

### Environmental modifiers of RNA splicing metabolism

An initial study into environmental factors that influence RNA splicing found that pre-mRNA splicing was blocked in heat shocked cells, suggesting that this posttranscriptional process was sensitive to cellular stress (Yost and Lindquist 1986). More recently, we showed that exposure to the heavy metal cadmium can also impact alternative splicing, indicating that environmental chemicals are potential modifiers of RNA splicing metabolism. Through screening the ToxCast chemical library in this study, we identified pararosaniline as a new chemical modifier of RNA splicing, and exposure to this chemical is deleterious to the development, reproduction, and aging of *C. elegans*.

At the cellular level, our gene expression analysis showed that pararosaniline induced oxidative stress as evidenced by the upregulation of a wide range of genes functioning in xenobiotic detoxification. A majority of the genes measured are controlled by the SKN-1/Nrf-2 transcription factor that is activated in response to pro-oxidant insults that induce oxidative stress (An and Blackwell 2003; Wu et al. 2016). It has been demonstrated that RNA splicing fidelity is sensitive to oxidative stress and that excess accumulation of reactive oxygen species can lead to deregulated alternative splicing (Disher and Skandalis 2007; Seo et al. 2016; Fontana et al. 2017). It is possible that, mechanistically, pararosaniline alters RNA splicing in *C. elegans* via induction of oxidative stress. This could suggest that pararosaniline influences RNA splicing in a nonspecific manner but rather as a consequence of impaired redox balance. Alternatively, we also observed that pararosaniline downregulated the expression of *snr-2*, which encodes a core building block of the spliceosome complex. In a mutant mouse that is heterozygous for *Snrpb* deletion (homolog of *snr-2*) where the expression of *Snrpb* is downregulated, there was a widespread increase in differentially spliced events. Notably, the most abundant type of aberrant splicing was exon skipping (Alam et al. 2022). We determined in this study that pararosaniline caused a downregulation to the *snr-2* mRNA levels and that transgenic overexpression of *snr-2* can provide partial resistance against pararosaniline-induced alterations to the splicing reporter, suggesting that this chemical may exert its toxicity in part by disrupting *snr-2* expression.

### Protective effects of translation suppression

Inhibition of protein translation has been shown to extend lifespan and enhance stress resistance of multiple organisms including worm, yeast, and fly (Kapahi et al. 2004; Hansen et al. 2007; Pan et al. 2007; Steffen et al. 2008; Zid et al. 2009). In *C. elegans*, the pro-longevity effect of translation suppression is in part mediated by the transcriptional upregulation of various stress response genes that directly enhance cellular resistance (Rogers et al. 2011). Translation suppression by inactivation of the eukaryotic initiation factor 4G (*ifg-1*) also inhibits cadmium-induced alternative splicing, potentially by enhancing the expression of more than 80 genes, including *snr-2*, that function in RNA metabolism (Chomyshen et al. 2022). In our targeted RNAi screen performed in this study, we observed that the knockdown of *ifg-1*, as well as genes encoding ribosomal subunits, also protects against pararosaniline's effect on RNA splicing in a similar manner as cadmium. This suggests that enhancing the expression of RNA splicing regulatory genes may serve as a protective mechanism against environmental stress-induced disruption to RNA splicing metabolism. While in this study we showed that overexpression of *snr-2* can partially provide resistance against pararosaniline-induced alternative splicing, the causality of different splicing factor gene expression with respect to resistance toward stress-induced splicing alterations remains to be individually investigated.

### Splicing fidelity as a marker for fitness

We observed that gene knockdowns that maintain GFP fluorescence of the splicing reporter after pararosaniline exposure are also protective against toxicity of this chemical on *C. elegans* survival. This could indicate that maintaining splicing fidelity under stress is positively associated with *C. elegans* survival capability. This correlation between splicing fidelity and fitness was also previously described when Heintz *et al.* (2017) found that worms that maintained high GFP fluorescence of the splicing reporter at 6 days old lived significantly longer than those with reduced GFP signal. There is a growing body of evidence that widespread alternative splicing events are found during aging and in chronic diseases including neurodegeneration and cancer (Tollervey et al. 2011; Mazin et al. 2013; Bonnal et al. 2020). While it remains a challenge to determine whether these splicing events are adaptive responses or have aberrant consequences, the utility of in vivo splicing reporter such as one employed in this study provides a unique opportunity for future investigation to identify small molecule compounds that can modulate splicing activity as a mean of interventions to promote organism fitness.

## Supplementary Material

jkad241_Supplementary_Data

## Data Availability

All datasets supporting this manuscript are presented within the article and supplementary files. Strains are available upon request.

## References

[jkad241-B1] Alam SS , Kumar S, Beauchamp MC, Bareke E, Boucher A, Nzirorera N, Dong Y, Padilla R, Zhang SJ, Majewski J et al 2022. Snrpb is required in murine neural crest cells for proper splicing and craniofacial morphogenesis. DMM Dis Model Mech. 15:dmm049544. doi:10.1242/dmm.049544. 35593225 PMC9235875

[jkad241-B2] An JH , Blackwell TK. 2003. SKN-1 links *C. elegans* mesendodermal specification to a conserved oxidative stress response. Genes Dev. 17(15):1882–1893. doi:10.1101/gad.1107803. 12869585 PMC196237

[jkad241-B3] Bhadra M , Howell P, Dutta S, Heintz C, Mair WB. 2020. Alternative splicing in aging and longevity. Hum Genet. 139(3):357–369. doi:10.1007/s00439-019-02094-6. 31834493 PMC8176884

[jkad241-B4] Bonnal SC , López-Oreja I, Valcárcel J. Roles and mechanisms of alternative splicing in cancer—implications for care. Nat Rev Clin Oncol. 2020;17(8):457–474. 32303702 10.1038/s41571-020-0350-x

[jkad241-B5] Brenner S . 1974. The genetics of *Caenorhabditis elegans*. Genetics. 77(1):71–94. doi:10.1093/genetics/77.1.71. 4366476 PMC1213120

[jkad241-B6] Chomyshen SC , Tabarraei H, Wu CW. 2022. Translational suppression via IFG-1/eIF4G inhibits stress-induced RNA alternative splicing in *Caenorhabditis elegans*. Genetics. 221(3):iyac075. doi:10.1093/genetics/iyac075. 35536193 PMC9252287

[jkad241-B7] Disher K , Skandalis A. 2007. Evidence of the modulation of mRNA splicing fidelity in humans by oxidative stress and p53. Genome. 50(10):946–953. doi:10.1139/G07-074. 18059557

[jkad241-B8] Fontana GA , Rigamonti A, Lenzken SC, Filosa G, Alvarez R, Calogero R, Bianchi ME, Barabino SML, et al 2017. Oxidative stress controls the choice of alternative last exons via a Brahma-BRCA1-CstF pathway. Nucleic Acids Res. 45(2):902–914. doi:10.1093/nar/gkw780. 27591253 PMC5314785

[jkad241-B9] Han SK , Lee D, Lee H, Kim D, Son HG, Yang J-S, Lee S-JV, Kim S, et al 2016. OASIS 2: online application for survival analysis 2 with features for the analysis of maximal lifespan and healthspan in aging research. Oncotarget. 7(35):56147–56152. doi:10.18632/oncotarget.11269. 27528229 PMC5302902

[jkad241-B10] Hansen M , Taubert S, Crawford D, Libina N, Lee S-J, Kenyon C. 2007. Lifespan extension by conditions that inhibit translation in *Caenorhabditis elegans*. Aging Cell. 6(1):95–110. doi:10.1111/j.1474-9726.2006.00267.x. 17266679

[jkad241-B11] Heintz C , Doktor TK, Lanjuin A, Escoubas CC, Zhang Y, Weir HJ, Dutta S, Silva-García CG, Bruun GH, Morantte I, et al 2017. Splicing factor 1 modulates dietary restriction and TORC1 pathway longevity in *C. elegans*. Nature. 541(7635):102–106. doi:10.1038/nature20789. 27919065 PMC5361225

[jkad241-B12] Huff JE , Haseman JK, DeMarini DM, Eustis S, Maronpot RR, Peters AC, Persing RL, Chrisp CE, Jacobs AC, et al 1989. Multiple-Site carcinogenicity of benzene in Fischer 344 rats and B6C3F1 mice. Environ. Health Perspect. 82:125–163. doi:10.1289/ehp.8982125. 2676495 PMC1568117

[jkad241-B13] Kapahi P , Zid BM, Harper T, Koslover D, Sapin V, Benzer S. 2004. Regulation of lifespan in *Drosophila* by modulation of genes in the TOR signaling pathway. Curr Biol. 14(10):885–890. doi:10.1016/j.cub.2004.03.059. 15186745 PMC2754830

[jkad241-B14] Kowalska J , Jeżewska A. 2019. Determination of pararosaniline hydrochloride in workplace air. Environ Monit Assess. 191(7):1–9. doi:10.1007/s10661-019-7568-z. PMC6579866 31209660

[jkad241-B15] Kuroyanagi H , Watanabe Y, Suzuki Y, Hagiwara M. 2013. Position-dependent and neuron-specific splicing regulation by the CELF family RNA-binding protein UNC-75 in *Caenorhabditis elegans*. Nucleic Acids Res. 41(7):4015–4025. doi:10.1093/nar/gkt097. 23416545 PMC3627589

[jkad241-B16] Mazin P , Xiong J, Liu X, Yan Z, Zhang X, Li M, He L, Somel M, Yuan Y, Phoebe Chen Y, et al 2013. Widespread splicing changes in human brain development and aging. Mol Syst Biol. 9(1):633. doi:10.1038/msb.2012.67. 23340839 PMC3564255

[jkad241-B17] McGhee JD , Sleumer MC, Bilenky M, Wong K, McKay SJ, Goszczynski B, Tian H, Krich ND, Khattra J, Holt RA, et al 2007. The ELT-2 GATA-factor and the global regulation of transcription in the *C. elegans* intestine. Dev Biol. 302(2):627–645. doi:10.1016/j.ydbio.2006.10.024. 17113066

[jkad241-B18] Montes M , Sanford BL, Comiskey DF, Chandler DS. 2019. RNA splicing and disease: animal models to therapies. Trends Genet. 35(1):68–87. doi:10.1016/j.tig.2018.10.002. 30466729 PMC6339821

[jkad241-B19] Nilsen TW . 2003. The spliceosome: the most complex macromolecular machine in the cell? BioEssays. 25(12):1147–1149. doi:10.1002/bies.10394. 14635248

[jkad241-B20] Nilsen TW , Graveley BR. 2010. Expansion of the eukaryotic proteome by alternative splicing. Nature. 463(7280):457–463. doi:10.1038/nature08909. 20110989 PMC3443858

[jkad241-B21] Pan KZ , Palter JE, Rogers AN, Olsen A, Chen D, Lithgow GJ, Kapahi P. 2007. Inhibition of mRNA translation extends lifespan in *Caenorhabditis elegans*. Aging Cell. 6(1):111–119. doi:10.1111/j.1474-9726.2006.00266.x. 17266680 PMC2745345

[jkad241-B22] Richard AM , Judson RS, Houck KA, Grulke CM, Volarath P, Thillainadarajah I, Yang C, Rathman J, Martin MT, Wambaugh JF, et al 2016. Toxcast chemical landscape: paving the road to 21st century toxicology. Chem Res Toxicol. 29(8):1225–1251. doi:10.1021/acs.chemrestox.6b00135. 27367298

[jkad241-B23] Rogers A , Chen D, McColl G, Czerwieniec G, Felkey K, Gibson B, Hubbard A, Melov S, Lithgow G, Kapahi P, et al 2011. Life span extension via eIF4G inhibition is mediated by posttranscriptional remodeling of stress response gene expression in *C. elegans*. Cell Metab. 14(1):55–66. doi:10.1016/j.cmet.2011.05.010. 21723504 PMC3220185

[jkad241-B24] Scotti MM , Swanson MS. 2015. RNA mis-splicing in disease. Nat Rev Genet. 17(1):19–32. doi:10.1038/nrg.2015.3. 26593421 PMC5993438

[jkad241-B25] Seo J , Singh NN, Ottesen EW, Sivanesan S, Shishimorova M, Singh RN. 2016. Oxidative stress triggers body-wide skipping of multiple exons of the spinal muscular atrophy gene. PLoS One. 11(4):e0154390. doi:10.1371/journal.pone.0154390. 27111068 PMC4844106

[jkad241-B26] Shetty S , Baig N, Bargakshatriya R, Pramanik K, Alameddine B. 2023. High uptake of the carcinogenic pararosaniline hydrochloride dye from water using carbazole-containing conjugated copolymers synthesized from a one-pot cyclopentannulation reaction. ACS Appl Mater Interfaces. 15(23):28149–28157. doi:10.1021/acsami.3c05639. 37257132

[jkad241-B27] Shin N , Cuenca L, Karthikraj R, Kannan K, Colaiácovo MP. 2019. Assessing effects of germline exposure to environmental toxicants by high-throughput screening in *C. elegans*. PLOS Genet. 15(2):e1007975. doi:10.1371/journal.pgen.1007975. 30763314 PMC6375566

[jkad241-B28] Steffen KK , MacKay VL, Kerr EO, Tsuchiya M, Hu D, Fox LA, Dang N, Johnston ED, Oakes JA, Tchao BN, et al 2008. Yeast life span extension by depletion of 60S ribosomal subunits is mediated by Gcn4. Cell. 133(2):292–302. doi:10.1016/j.cell.2008.02.037. 18423200 PMC2749658

[jkad241-B29] Tabarraei H , Waddell BM, Raymond K, Murray SM, Wang Y, Choe KP, Wu C. 2023. CCR4-NOT subunit CCF-1/CNOT7 promotes transcriptional activation to multiple stress responses in *Caenorhabditis elegans*. Aging Cell. 22(4):e13795. doi:10.1111/acel.13795. 36797658 PMC10086529

[jkad241-B30] Tkaczyk-Wlizło A , Mitrowska K, Błądek T. 2022. Quantification of twenty pharmacologically active dyes in water samples using UPLC-MS/MS. Heliyon. 8(4):e09331. doi:10.1016/j.heliyon.2022.e09331. 35520618 PMC9062210

[jkad241-B31] Tollervey JR , Wang Z, Hortobágyi T, Witten JT, Zarnack K, Kayikci M, Clark TA, Schweitzer AC, Rot G, Curk T, et al 2011. Analysis of alternative splicing associated with aging and neurodegeneration in the human brain. Genome Res. 21(10):1572. doi:10.1101/gr.122226.111. 21846794 PMC3202275

[jkad241-B32] Will CL , Lührmann R. 2011. Spliceosome structure and function. Cold Spring Harb. Perspect. Biol. 3(7):pii: a003707. doi:10.1101/cshperspect.a003707. PMC3119917 21441581

[jkad241-B33] Wu CW , Deonarine A, Przybysz A, Strange K, Choe KP. 2016. The Skp1 homologs SKR-1/2 are required for the *Caenorhabditis elegans* SKN-1 antioxidant/detoxification response independently of p38 MAPK. PLoS Genet. 12(10):e1006361. doi:10.1371/journal.pgen.1006361. 27776126 PMC5077136

[jkad241-B34] Wu CW , Wimberly K, Pietras A, Dodd W, Atlas MB, Choe KP, et al 2019. RNA processing errors triggered by cadmium and integrator complex disruption are signals for environmental stress. BMC Biol. 17(1):56. doi:10.1186/s12915-019-0675-z. 31311534 PMC6631800

[jkad241-B35] Yost HJ , Lindquist S. 1986. RNA splicing is interrupted by heat shock and is rescued by heat shock protein synthesis. Cell. 45(2):185–193. doi:10.1016/0092-8674(86)90382-X. 2421918

[jkad241-B36] Zid BM , Rogers AN, Katewa SD, Vargas MA, Kolipinski MC, Lu TA, Benzer S, Kapahi P, et al 2009. 4E-BP extends lifespan upon dietary restriction by enhancing mitochondrial activity in *Drosophila*. Cell. 139(1):149–160. doi:10.1016/j.cell.2009.07.034. 19804760 PMC2759400

