# Supplementary material for: Identification of pararosaniline as a modifier of RNA splicing in Caenorhabditis elegans
Source: G3 (Bethesda). 2023 Oct 19;13(12):jkad241. doi: 10.1093/g3journal/jkad241 (PMC10700105; doi:10.1093/g3journal/jkad241)
Supplement: jkad241_Supplementary_Data [file jkad241_supplementary_data.zip › Figure_S2_G3-2023-404511.pdf]

a

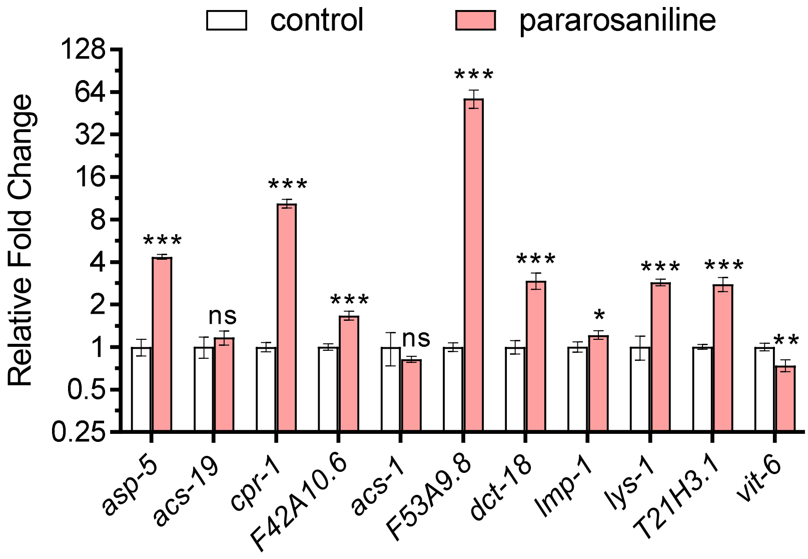

b

Gene	Description
asp-5	Aspartyl protease; predicted aspartic endopeptidase activity
acs-19	Fatty acid CoA synthetase; predicted acetate-CoA ligase activity
cpr-1	Cysteine protease related; predicted cysteine endopeptidase activity
F42A10.6	No characterized function
acs-1	Fatty acid CoA synthetase; predicted medium-chain fatty acid-CoA ligase activity
F53A9.8	Involved in defense to gram-positive bacterium
dct-18	DAF-16/FOXO controlled, germline Tumor affecting
lmp-1	Lysosome-associated membrane protein
lys-1	Lysozyme; involved in defense to gram-negative bacterium
T21H3.1	Involved in lipid metabolic process
vit-6	Vitellogenin structural gene; enable lipid transporter activity
