# Supplementary material for: Identification of pararosaniline as a modifier of RNA splicing in Caenorhabditis elegans
Source: G3 (Bethesda). 2023 Oct 19;13(12):jkad241. doi: 10.1093/g3journal/jkad241 (PMC10700105; doi:10.1093/g3journal/jkad241)
Supplement: jkad241_Supplementary_Data [file jkad241_supplementary_data.zip › Supplemental_Figure_Legends_G3-2023-404511.docx]

**Figure S1**. Effect of pararosaniline on an inverted splicing reporter. **a**. Schematic of the inverted *in vivo* RNA splicing reporter **b**. Representative fluorescent micrograph and **c**. quantification of the inverted splicing reporter after treatment with 0 – 2 mM of pararosaniline. The data point in **c** indicates mean ± SD of N = 3 groups of 8 worms/group. * P<0.05 and ** P<0.01 as determine by One-way ANOVA.

**Figure S2**. Effects of pararosaniline exposure on intestinal specific gene expression. **a**. Relative expression and **b**. description of intestinal specific genes after exposure to pararosaniline. *P<0.05, **P<0.01, and ***P<0.001 as determined by the student’s t-test corrected for multiple comparisons using the Holm-Sidak method.

**Table S1**. Primers used in this study

**Table S2**. List of chemical hits

**Table S3**. Lifespan assay statistics
