# Supplementary figures and images for: Identification of pararosaniline as a modifier of RNA splicing in Caenorhabditis elegans
Source: G3 (Bethesda). 2023 Oct 19;13(12):jkad241. doi: 10.1093/g3journal/jkad241 (PMC10700105; doi:10.1093/g3journal/jkad241)

**a****Inverted splicing reporter (WBM535)**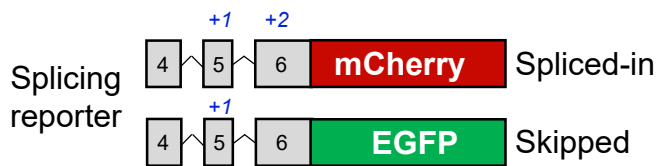**b**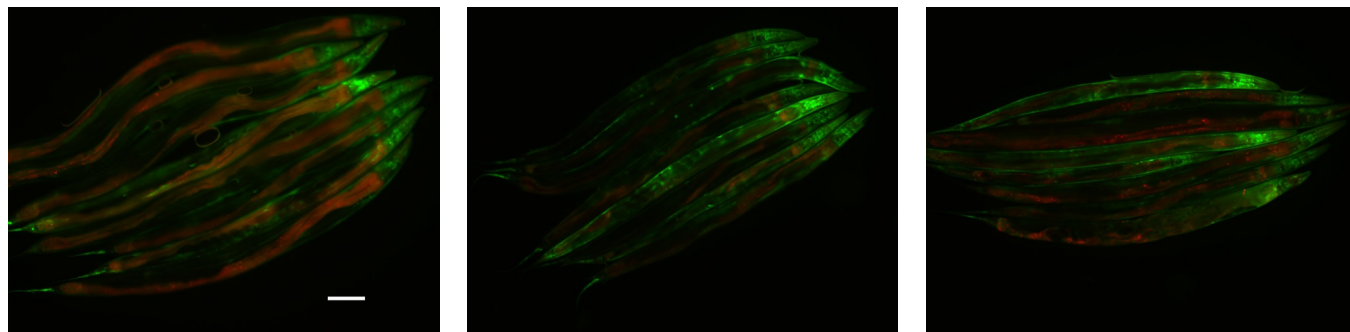

0 mM pararosaniline

1 mM pararosaniline

2 mM pararosaniline

**c**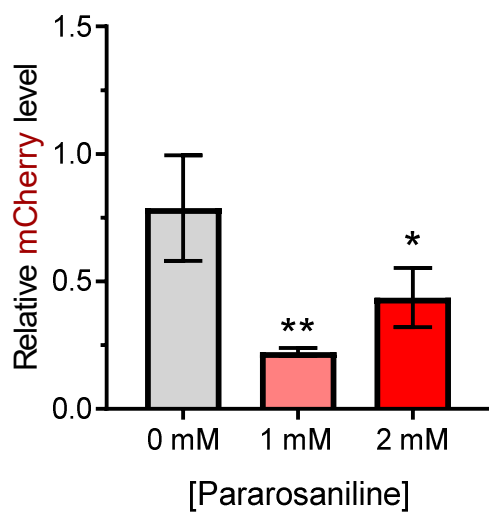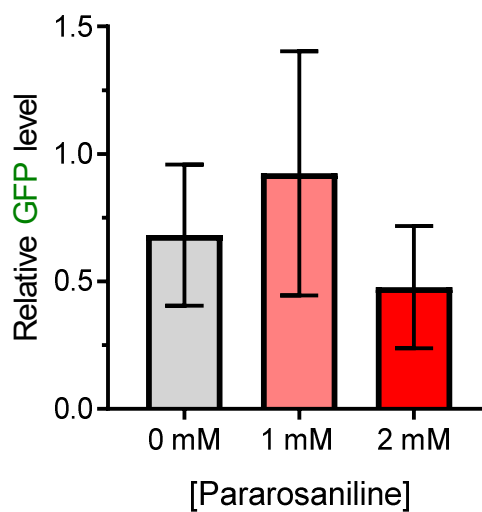

Supplement: jkad241_Supplementary_Data [file jkad241_supplementary_data.zip › Figure_S1_G3-2023-404511.pdf]
